# The effect of clustering on lot quality assurance sampling: a probabilistic model to calculate sample sizes for quality assessments

**DOI:** 10.1186/1742-7622-10-11

**Published:** 2013-10-26

**Authors:** Bethany L Hedt-Gauthier, Tisha Mitsunaga, Lauren Hund, Casey Olives, Marcello Pagano

**Affiliations:** 1 Department of Global Health and Social Medicine, Harvard Medical School, Boston, USA; 2 Inshuti Mu Buzima (IMB)/Partners In Health (PIH), Rwinkwavu, Rwanda; 3 Department of Biostatistics, Harvard School of Public Health, Boston, USA; 4 Department of Family and Community Medicine, University of New Mexico, Albuquerque, New Mexico, USA; 5 Department of Biostatistics, University of Washington, Seattle, USA

**Keywords:** Cluster-LQAS, Lot quality assurance sampling, Program evaluation, Survey, Community health workers

## Abstract

**Background:**

Traditional Lot Quality Assurance Sampling (LQAS) designs assume observations are collected using simple random sampling. Alternatively, randomly sampling clusters of observations and then individuals within clusters reduces costs but decreases the precision of the classifications. In this paper, we develop a general framework for designing the cluster(C)-LQAS system and illustrate the method with the design of data quality assessments for the community health worker program in Rwanda.

**Results:**

To determine sample size and decision rules for C-LQAS, we use the beta-binomial distribution to account for inflated risk of errors introduced by sampling clusters at the first stage. We present general theory and code for sample size calculations.

The C-LQAS sample sizes provided in this paper constrain misclassification risks below user-specified limits. Multiple C-LQAS systems meet the specified risk requirements, but numerous considerations, including per-cluster versus per-individual sampling costs, help identify optimal systems for distinct applications.

**Conclusions:**

We show the utility of C-LQAS for data quality assessments, but the method generalizes to numerous applications. This paper provides the necessary technical detail and supplemental code to support the design of C-LQAS for specific programs.

## Background

Lot quality assurance sampling (LQAS) was originally developed as an industrial control tool [1]. From a lot of goods, a random sample of *n* items are selected and inspected for defects. If a certain number, *d*, or more goods have a defect, then the lot is rejected from distribution and the industrial process reviewed to address the problem. If fewer then *d* defects are detected, then the lot is determined to be of acceptable quality and shipped. In the 1980s, LQAS emerged as a useful tool in public health to identify areas with suboptimal program coverage or unacceptable levels of disease [2-6].

The binomial model, assuming simple random sampling, is ordinarily used to determine the LQAS design when dealing with large populations. This approach allows for the calculation of both the required sample size, n, and the decision rule, d. These two design parameters are governed by four parameters: 1) the upper threshold, *p*_*u*_ — the proportion of defective items at or above which a lot from such a population should be classified as suboptimal; 2) the maximum allowable risk of misclassification error at the upper threshold, *α*_*max*_; 3) the lower threshold, *p*_*l*_ – the proportion of defective items, at or below which a lot should be classified to have sufficient quality; and 4) the maximum allowable risk of misclassification error at the lower threshold, *β*_*max*_. The requisite relationships are:

$$P\left(X<d|n,p_{u}\right)\leq \alpha_{\max}$$

and

$$P\left(X\geq d|n,p_{l}\right)\leq \beta_{\text{max}},$$

where *X* is a binomial random variable representing the number of 'defects’ observed in the sample of size n, with probability of observing a defect set to p_l_ or p_u_.

Sometimes a simple random sample is not available—for example, geographic spread may make a simple random sample infeasible [7-9]— and one must resort to other sampling designs. One such instance is the data quality assessment of the community health worker (CHW) program in southern Kayonza, Rwanda. For this program supported by Partners In Health, CHWs visit their assigned households monthly to document key demographic and health indicators. These data are used to manage CHW activities, forecast health needs in the area, and evaluate program implementation. Thus, ensuring high quality CHW data is important.

Each CHW is responsible for 40–60 households, with 2–4 CHWs per village, and about seven villages per cell [10]. Since CHWs are supervised at the cell level, we are interested in classifying the data quality for each cell. Cells identified as having low data quality would receive additional support, such as extra training for the CHWs and the cell supervisor. Cells identified as having sufficiently high quality data would receive some positive reinforcement, such as certificates of achievement.

Applying the traditional LQAS system, we would randomly sample *n* households from the approximately 1000 households in a cell. Each household would be visited and re-measured. The new data would then be compared to the data documented in the CHW household register. If *d* or more households captured in the CHW registers have discrepant data, then the cell is classified as having insufficient quality. If fewer then *d* households have discrepant data, then the cell is classified as high data quality. The challenge, however, is the potential for large geographic spread of the sampled households requiring an intensive amount of resources and time. In the most extreme case, the *n* households could come from *n* different and widespread villages. To lighten the burden on the program by providing a more concentrated sample, we explore a two stage cluster-sampling procedure: first randomly sample a number of CHWs, and second sample households within these CHWs’ domain.

This method of sampling lowers the resources required for the data quality assessments, as sampling households in the same village requires significantly fewer resources than sampling households in different villages. However, previous work has shown that the implementation of an LQAS system in such a clustered manner when *n* and *d* are incorrectly calculated under the assumption of simple random sampling raises the misclassification risks above the specified bounds because of the correlation within clusters [7,8,11,12]. In 2010, Pezzoli et al. describe an approach for developing a cluster-LQAS (C-LQAS) system where the total sample size, *n*, is based on the binomial distribution [12]. They divide the total sample size into *m* clusters, each of size *k* and then estimate the true error rates of the C-LQAS systems (adjusting for correlation) via simulations. Their approach for designing the C-LQAS system has several limitations. The method calculates errors for a specific system and determines whether these errors are sufficiently low instead of designing a system to restrict misclassification risks below program-specified bounds. Restricting systems to those with a total sample size of *n* provides a limited number of options, and it is possible that for certain conditions, none of the designs will have misclassification risks below desired levels.

In this paper, we provide a general probability framework for determining the appropriate sample size for a C-LQAS system that meets the desired constraints without restricting it to systems with a fixed total sample size of *n*. The theory for C-LQAS is presented in the methods section. In the results section, we illustrate the method by calculating the sample sizes and decision rules for the CHW data quality assessment in Rwanda and present sensitivity analyses using simulation results to assess the performance of the selected C-LQAS sampling designs when the assumptions used to develop the system are not fully met. Finally, the discussion presents considerations for designing C-LQAS systems for other contexts.

## Methods

### Calculating sample sizes and decision rules for cluster-LQAS

Basic two-way LQAS aims to classify an area into one of two categories – defective or acceptable quality if referring to a lot of goods, high or low performance if referring to program coverage. The sample size, *n*, and decision rule, *d*, are determined by the upper and lower thresholds (*p*_*u*_ and *p*_*l*_) and the maximum allowable misclassification risks at each threshold (*α*_*max*_ and *β*_*max*_, respectively). These parameters are set *a priori* by program managers and are determined by a variety of factors. The thresholds can be determined by program targets, key epidemiological parameters, or knowledge of underlying distribution. The maximum misclassification risks reflect the amount of uncertainty tolerable in an evaluation with consideration of the resulting consequences of failing to allot resources to areas in need or to spread limited resources too thin by allocating additional resources to high performing areas.

For basic two-way LQAS actual probability of a certain classification for a given *p* is based on the binomial model. Areas at or above the upper threshold have a high probability (≥1- *α*_*max*_) of being correctly classified in the upper category (in this case, being classified as defective) and areas at or below the lower threshold have a high probability (≥1- *β*_*max*_) of being classified in the lower category (in this case, being classified as of acceptable quality). The area between the two thresholds, often referred to as the *grey area,* has less discernible classification properties. Typically in the grey area, the closer to a threshold, the more likely to be classified into the corresponding category though in the most extreme, areas in the between the upper and lower thresholds can have a 50–50 chance of being classified into a given category.

The basic two-way LQAS system is defined by two numbers: *n*, the number of items randomly sampled from the lot and the decision rule, *d*. If *d* or more of the *n* items are defective, then the lot is rejected. Otherwise, the lot is classified to be of acceptable quality. The design of a C-LQAS system is defined by three numbers: the number of clusters sampled, *m*; the number of items per cluster sampled, *k*; and the decision rule for classification, *d*[7,8]. The number of defective 'units’ across the m*k elements are tallied and compared to the decision rule in the same way as the basic two-way LQAS. If *d* or more are defective, then the lot is rejected; if fewer than *d* are defective, then the lot is classified to be of acceptable quality.

If there is no correlation in the presence or absence of defects within clusters, then calculating the sample size, n = m*k, and the decision rule based on the binomial distribution assuming simple random sampling is sufficient to maintain the risk of errors below specified levels. However, if the sample size is calculated using the binomial model that assumes simple random sampling but there is clustering of the outcome, then the true misclassification risks are likely greater than the specified limits, α_*max*_ and β_*max*_[7,8,11,12].

To overcome this problem, we propose using the beta-binomial model to incorporate the clustering effect into the sample size and decision rule calculations. In addition to specifying the four parameters above (p_l_, p_u_, α_*max*_ and β_*max*_), one must also specify ρ, the intraclass correlation (ICC). Reliable estimates of ICC are critical for obtaining appropriate designs and more detail on this topic is presented in the discussion.

Based on these five parameters, the program manager finds a recommended number of clusters and number of individuals per cluster to sample, along with a corresponding decision rule, that reduces the risk of misclassification below the specified levels (Additional file 1). The methods and results presented assume that the population sizes within clusters are equal. The risk limits are not always achievable. Even for infinitely large samples in a cluster, we must sample at least ρ* *n*_*min*_ clusters, where *n*_*min*_ is the minimum sample size needed for an LQAS system with no clustering, to meet the specified constraints for the C-LQAS system. For a given ρ, and any m > ρ* *n*_*min*_, there are multiple k to satisfy the desired risk profile. We choose the smallest such k.

We provide code developed for R version 2.15.1 that calls the VGAM package [13] for the probability distribution function of the beta-binomial to calculate the number of samples per cluster and the corresponding decision rules for a range of number of clusters, *m*, up to *m = n*_*min*_ (Additional file 2).

### Application to the CHW data quality assessment

To illustrate the use of this method, we apply the above theory to calculate the sample size for the CHW data quality assessment in Rwanda. The aim of the data quality assessment is to monitor, at the cell level, the accuracy of household data collected by CHWs. For each cell, using the C-LQAS system, *m* CHWs and *k* households per CHW are randomly chosen. At each sampled household, information collected during a supervision visit of the household are compared to the information in the CHW register. As an example, we consider just one data element collected by CHWs – whether the number of children (under five years old) in the household is correctly documented in the CHW register.

One possible approach to selecting a particular C-LQAS design for a set of parameters is to introduce a cost function into the decision making. Consider the per cluster and per individual costs, *C*_*m*_ and *C*_*k*_ respectively, and define the total cost for the implementation, *C*_*total*_ *= C*_m_*m + C_k_*m*k. The total costs for C-LQAS designs under different assumed per cluster and per individual costs are provided.

### Simulation of performance of cluster-LQAS

Using simulations we evaluated the performance of various C-LQAS systems. The results are based on 10,000 simulation draws from a beta-binomial distribution for a given set of values of m, k, ρ, and p. The first set of simulations looked at the effect of misspecification of ICC in the design on the misclassification errors. The second set of simulations also evaluated the misclassification errors for misspecification of ICC as well as the mean and standard error of the ICC using the Analysis of Variance (ANOVA) estimator [14]. For simulations that resulted in no events in any of the clusters, the ANOVA ICC is inestimable and we thus excluded them from the results. We report the percent of simulations with valid ANOVA estimates.

## Results

### Application to the CHW data quality assessment

For the design of the C-LQAS system for the data quality assessment in Rwanda, the following parameters are specified:

1. p_u_ = 0.25 – cells with 25% or higher error rates in documentation of the number of children are considered to have insufficient data quality;

2. α_*max*_ = 0.10 – restrict the probability of classifying cells with 25% or higher error rates in documentation as sufficient data quality to less than 10%;

3. p_l_ = 0.05 – cells with 5% or lower error rates in documentation of the number of children are considered to have sufficient data quality;

4. β_*max*_ = 0.10 – restrict the probability of classifying cells with 5% or lower error rates in documentation as insufficient data quality to less than 10%; and

5. ρ = 0.1 – the ICC describing the amount that data quality clusters by CHW.

Using these parameter values, and ignoring the clustered nature of the sampling, the recommended sample size is 20 with d = 3. The expected probability of misclassification of such a system, based on the (non-clustered) binomial distribution are α = 0.091 and β = 0.075. Table 1 presents the recommended sample sizes and expected misclassification errors for a range of ICCs (ρ = 0.01, 0.025, 0.05, 0.1, 0.15, and 0.2) and number of clusters (m = 2-20) to meet the constraints specified above. When ρ = 0.1, total sample sizes range from 20 (*m* = 20, *k* = 1) to 136 (*m* = 2, *k* = 68). For small number clusters sampled and large ICC, some of the designs may not be suitable for the CHW data quality assessment as the recommended number of samples per cluster exceed the average number of households available in a village. For a fixed *m*, the per cluster sample size increases as the ICC increases. As a general rule, for a fixed ICC, the total sample size increases as the number of clusters sampled decreases. This rule may not hold when the sample sizes are small and the requirement that all clusters be of the same size is imposed because of the discrete nature of the beta-binomial model.

**Table 1:** **Recommended sample sizes and decision rules for numbers of clusters up to n**_**min **_**for the community health worker data quality assessment example**

**Number of clusters**																				
		**2**	**3**	**4**	**5**	**6**	**7**	**8**	**9**	**10**	**11**	**12**	**13**	**14**	**15**	**16**	**17**	**18**	**19**	**20**
ICC = 0.01	samples per cluster, k	13	7	5	4	5	3	4	3	2	2	3	2	2	2	2	2	2	2	1
	total sample size, n	26	21	20	20	30	21	32	27	20	22	36	26	28	30	32	34	36	38	20
	decision rule, d	4	3	3	3	4	3	5	4	3	3	5	4	4	4	5	5	5	5	3
	α	0.093	0.081	0.096	0.095	0.040	0.077	0.073	0.069	0.092	0.062	0.036	0.081	0.056	0.038	0.071	0.050	0.035	0.024	0.091
	β	0.048	0.091	0.079	0.078	0.065	0.087	0.022	0.045	0.076	0.096	0.034	0.040	0.050	0.062	0.021	0.027	0.033	0.041	0.075
	Expected Costs, Scenario 1 †	$1,260	$1,710	$2,200	$2,700	$3,300	$3,710	$4,320	$4,770	$5,200	$5,720	$6,360	$6,760	$7,280	$7,800	$8,320	$8,840	$9,360	$9,880	$10,200
	Expected Costs, Scenario 2 ‡	$1,900	$1,950	$2,200	$2,500	$3,300	$3,150	$4,000	$4,050	$4,000	$4,400	$5,400	$5,200	$5,600	$6,000	$6,400	$6,800	$7,200	$7,600	$7,000
ICC = 0.025	samples per cluster, k	14	7	7	4	5	3	4	3	2	2	3	2	2	2	2	2	2	2	1
	total sample size, n	28	21	28	20	30	21	32	27	20	22	36	26	28	30	32	34	36	38	20
	decision rule, d	4	3	4	3	4	3	5	4	3	3	5	4	4	4	5	5	5	5	3
	α	0.084	0.090	0.069	0.100	0.045	0.080	0.077	0.072	0.094	0.063	0.038	0.083	0.057	0.039	0.072	0.051	0.036	0.025	0.091
	β	0.074	0.098	0.062	0.083	0.070	0.090	0.025	0.048	0.078	0.097	0.036	0.041	0.051	0.063	0.022	0.028	0.034	0.042	0.075
	Expected Costs, Scenario 1	$1,280	$1,710	$2,280	$2,700	$3,300	$3,710	$4,320	$4,770	$5,200	$5,720	$6,360	$6,760	$7,280	$7,800	$8,320	$8,840	$9,360	$9,880	$10,200
	Expected Costs, Scenario 2	$2,000	$1,950	$2,600	$2,500	$3,300	$3,150	$4,000	$4,050	$4,000	$4,400	$5,400	$5,200	$5,600	$6,000	$6,400	$6,800	$7,200	$7,600	$7,000
ICC = 0.05	samples per cluster, k	18	10	7	6	5	3	4	3	2	2	3	2	2	2	2	2	2	2	1
	total sample size, n	36	30	28	30	30	21	4	27	20	22	36	26	28	30	32	34	36	38	20
	decision rule, d	5	4	4	4	4	3	5	4	3	3	5	4	4	4	5	5	5	5	20
	α	0.093	0.071	0.082	0.056	0.052	0.085	0.085	0.076	0.097	0.065	0.041	0.086	0.060	0.041	0.075	0.053	0.038	0.026	0.091
	β	0.083	0.095	0.073	0.082	0.078	0.094	0.029	0.052	0.080	0.100	0.040	0.043	0.054	0.065	0.023	0.029	0.036	0.044	0.075
	Expected Costs, Scenario 1	$1,360	$1,800	$2,280	$2,800	$3,300	$3,710	$4,320	$4,770	$5,200	$5,720	$6,360	$6,760	$7,280	$7,800	$8,320	$8,840	$9,360	$9,880	$10,200
	Expected Costs, Scenario 2	$2,400	$2,400	$2,600	$3,000	$3,300	$3,150	$4,000	$4,050	$4,000	$4,400	$5,400	$5,200	$5,600	$6,000	$6,400	$6,800	$7,200	$7,600	$7,000
ICC = 0.1	samples per cluster, k	68‡‡	15	9	6	5	4	4	3	3	3	3	2	2	2	2	2	2	2	1
	total sample size, n	136	45	36	30	30	28	32	27	30	33	36	26	28	30	32	34	36	38	20
	decision rule, d	17	6	5	4	4	4	5	4	4	5	5	4	4	4	5	5	5	5	3
	α	0.096	0.092	0.090	0.075	0.067	0.082	0.099	0.086	0.052	0.077	0.048	0.091	0.064	0.045	0.080	0.058	0.041	0.029	0.091
	β	0.099	0.093	0.082	0.099	0.093	0.073	0.038	0.060	0.078	0.036	0.047	0.047	0.058	0.070	0.026	0.033	0.040	0.048	0.075
	Expected Costs, Scenario 1	$2,360	$1,950	$2,360	$2,800	$3,300	$3,780	$4,320	$4,770	$5,300	$5,830	$6,360	$6,760	$7,280	$7,800	$8,320	$8,840	$9,360	$9,880	$10,200
	Expected Costs, Scenario 2	$7,400	$3,150	$3,000	$3,000	$3,300	$3,500	$4,000	$4,050	$4,500	$4,950	$5,400	$5,200	$5,600	$6,000	$6,400	$6,800	$7,200	$7,600	$7,000
ICC = 0.15	samples per cluster, k	††	45‡‡	13	9	6	4	5	3	3	3	3	2	2	2	2	2	2	2	1
	total sample size, n		135	52	45	36	28	40	27	30	33	36	26	28	30	32	34	36	38	20
	decision rule, d		17	7	6	5	4	6	4	4	5	5	4	28	4	5	5	5	5	3
	α		0.099	0.100	0.082	0.087	0.095	0.090	0.096	0.060	0.086	0.055	0.096	0.069	0.049	0.085	0.062	0.045	0.032	0.091
	β		0.099	0.090	0.087	0.080	0.083	0.043	0.067	0.086	0.042	0.053	0.051	0.062	0.074	0.029	0.036	0.043	0.051	0.075
	Expected Costs, Scenario 1		$2,850	$2,520	$2,950	$3,360	$3,780	$4,400	$4,770	$5,300	5830.000	$6,360	$6,760	$7,280	$7,800	$8,320	$8,840	$9,360	$9,880	$10,200
	Expected Costs, Scenario 2		$7,650	$3,800	$3,750	$3,600	$3,500	$4,400	$4,050	$4,500	4950.000	$5,400	$5,200	$5,600	$6,000	$6,400	$6,800	$7,200	$7,600	$7,000
ICC = 0.2	samples per cluster, k	††	††	40	12	9	6	6	4	3	3	3	3	2	2	2	2	2	2	1
	total sample size, n			160	60	54	42	48	36	30	33	36	39	28	30	32	34	36	38	20
	decision rule, d			20	8	7	6	7	5	4	5	5	5	4	4	5	5	5	5	3
	α			0.096	0.099	0.077	0.098	0.091	0.077	0.068	0.095	0.063	0.041	0.073	0.052	0.090	0.066	0.048	0.035	0.091
	β			0.100	0.090	0.091	0.067	0.050	0.071	0.093	0.047	0.060	0.074	0.066	0.078	0.032	0.039	0.047	0.055	0.075
	Expected Costs, Scenario 1			$3,600	$3,100	$3,540	$3,920	$4,480	$4,860	$5,300	$5,830	$6,360	$6,890	$7,280	$7,800	$8,320	$8,840	$9,360	$9,880	$10,200
	Expected Costs, Scenario 2			$9,200	$4,500	$4,500	$4,200	$4,480	$4,500	$4,500	$4,950	$5,400	$5,850	$5,600	$6,000	$6,400	$6,800	$7,200	$7,600	$7,000

This table assumes *p*_*u*_ = 0.25, *p*_*l*_ = 0.05, α_*max*_ *=* β_*mas*_ = 0.1.

† Assuming costs of $500 per cluster and $10 per individual sampled. ‡ Assuming costs of $300 per cluster and $50 per individual sampled. †† Too few clusters to determine a system. ‡‡ May not be applicable to CHW problem, as recommended sample size exceeds average number of households per cluster.

In Table 1, we also present the estimated costs for each system under two possibilities, one with large per cluster costs (*C*_*m*_ = $500) and small per individual costs (*C*_*k*_ = $10) and the other with moderate per cluster costs (*C*_*m*_ = $300) and larger per individual costs (*C*_*k*_ = $50). These two disparate costing ratios (*C*_*m*_*/C*_*k*_), 50 to 1 and 6 to 1, cover a spectrum of possibilities. The least expensive C-LQAS for a given ICC depends on the costing ratio. For example, for ρ = 0.1, the design with {*m = 3, k = 15, d = 6*} is the least expensive ($1950) under the first cost structure and the designs with {*m = 4, k = 9, d = 5*} and {*m = 5, k = 6, d = 4*} are the cheapest ($3000) under the second cost structure.

### Simulation of performance of cluster-LQAS

**Figure 1:**
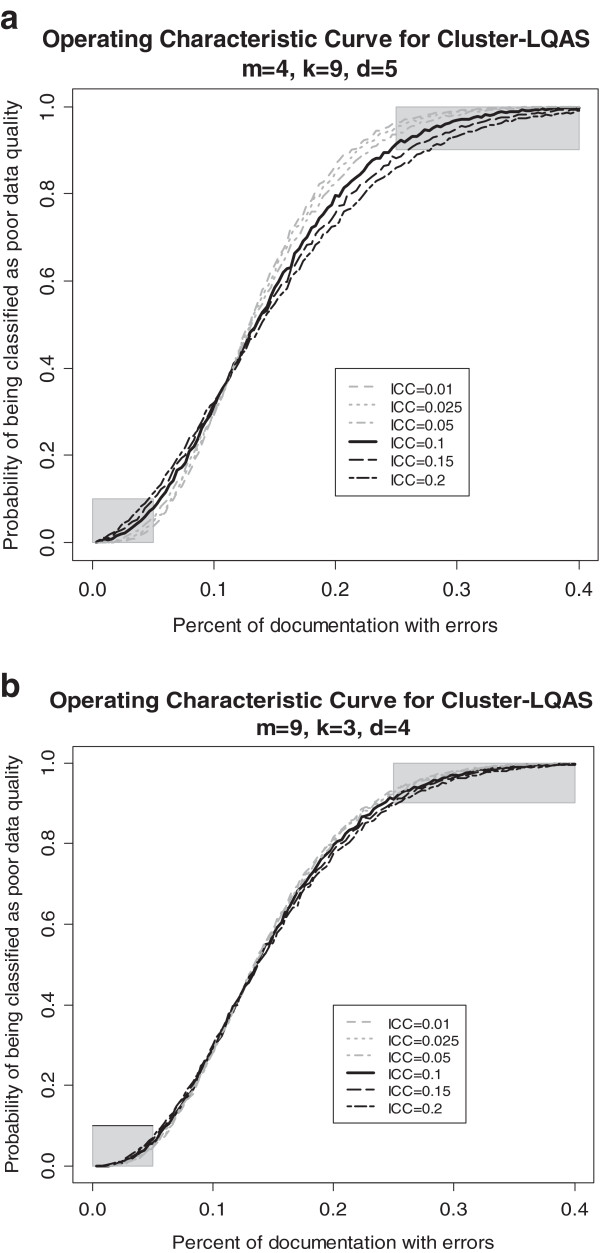
Operating Characteristic Curve for the C-LQAS system a) {m = 4, k = 9, d = 5} and b) {m = 9, k = 3, d = 4} under varying values of intraclass correlation (ICC).

To examine the impact of misspecification of ρ, we first evaluate two different C-LQAS systems for different values of ρ. The two designs are based on the parameters specified above for the CHW data quality assessment assuming ρ = 0.1 – namely, {m = 4,k = 9,d = 5} and {m = 9,k = 3,d = 4}. The effect of misspecifying ICC in the design on misclassification risks is evident in the Operating Characteristic Curves, the curve that fits the probability of classifying an area as poor data quality via a specific C-LQAS system for a range of proportions of true errors in data. As seen in Figure 1a and b, when the true ICC is equal to that specified in the design (in this case ρ = 0.1) the probability of classifying an area as having poor data quality remains below β_*max*_ = 0.1 when the proportion of registers with errors is less than 5% (line falls in the lower shaded box for *p <* 5%) and is above 0.9 (1-α_*max*_) when the proportion of registers with errors is greater than 25% (the line falls in the upper shaded box for *p <* 25%). However, when the ICC is above the value estimated for the design of the system, then the misclassification errors often exceed α_*max*_ and β_*max*_. Despite the bigger overall sample sizes, the systems with fewer sampled clusters (m = 4) are more likely to exceed α_*max*_ and β_*max*_ than the system with more sampled clusters (m = 9) when the true ICC is larger than expected, illustrating that, when cost is not an issue, sampling more clusters is a more conservative design. Any other ICC values smaller than the specified ρ = 0.1 result in systems with misclassification risks smaller than α_*max*_ and β_*max.*_

Next, we present three C-LQAS systems that satisfy the misclassification constraints for ρ = 0.01 (Table 2), ρ = 0.1 (Table 3), and ρ = 0.2 (Table 4). As above, when the ICC used for a set of simulations was equal to or less than the value assumed for designing the system, the observed α and β values were less than the constraints. With a true ICC larger than the value assumed in the design, the risks often exceeded the misclassification constraints. The more clusters in the sample, the smaller the impact of the ICC, and thus a lower risk of exceeding misclassification constraints even when the ICC is underestimated in the design.

**Table 2:** Observed misclassification errors and estimated intraclass correlation for varying values of intraclass correlation (ICC), assuming ICC = 0.01 in the design

			**From simulations at *p***_***l***_ **= 0.05**			**From simulations at *p***_***u***_ **= 0.25**			
**ρ**	**α**	**β**	**ICC, mean**	**ICC, standard deviation**	**Percent of simulations with observable ICC†**	**ICC, mean**	**ICC, standard deviation**	**Percent of simulations with observable ICC**	
m = 4, k = 5, d = 3									
*0.010*	*0.098*	*0.080*	*-0.007*	*0.095*	*0.633*	*-0.007*	*0.160*	*0.996*	
0.025	0.101	0.089	0.000	0.102	0.632	0.004	0.166	0.997	
0.050	0.113	0.095	0.015	0.119	0.612	0.025	0.179	0.993	
0.100	0.132	0.111	0.043	0.144	0.578	0.065		0.195	0.991
0.150	0.153	0.114	0.075	0.177	0.537	0.103	0.214	0.986	
0.200	0.173	0.127	0.100	0.200	0.524	0.148	0.236	0.981	
m = 7, k = 3, d = 3									
*0.010*	*0.074*	*0.087*	*0.086*	*0.121*	*0.644*	*0.083*	*0.226*	*0.997*	
0.025	0.083	0.094	0.098	0.141	0.648	0.095	0.231	0.999	
0.050	0.088	0.099	0.113	0.158	0.639	0.122	0.236	0.996	
0.100	0.097	0.110	0.142	0.189	0.623	0.167	0.246	0.995	
0.150	0.107	0.110	0.178	0.221	0.603	0.210	0.258	0.994	
0.200	0.117	0.117	0.211	0.250	0.591	0.256	0.266	0.993	
m = 9, k = 3, d = 4									
*0.010*	*0.069*	*0.044*	*0.103*	*0.126*	*0.747*	*0.099*	*0.203*	*1.000*	
0.025	0.071	0.047	0.112	0.136	0.739	0.114	0.208	0.999	
0.050	0.076	0.053	0.127	0.151	0.738	0.135	0.210	0.999	
0.100	0.083	0.061	0.160	0.188	0.725	0.184	0.224	0.999	
0.150	0.098	0.067	0.193	0.217	0.691	0.234	0.234	0.999	
0.200	0.108	0.073	0.228	0.242	0.681	0.278	0.239	0.998	

Intraclass correlation, † At least one or more events in at least one cluster. Italic data corresponds to the ICC used in the design.

**Table 3:** Observed misclassification errors and intraclass correlation for varying values of intraclass correlation (ICC), assuming ICC = 0.10 in the design

			**From simulations at p**_**l**_ **= 0.05**			**From simulations at p**_**u**_ **= 0.25**			
**ρ**	**α**	**β**	**ICC, mean**	**ICC, standard deviation**	**Percent of simulations with observable ICC†**	**ICC, mean**	**ICC, standard deviation**	**Percent of simulations with observable ICC**	
m = 4, k = 9, d = 5									
0.010	0.041	0.040	-0.011	0.060	0.830	-0.009	0.085	1.000	
0.025	0.049	0.051	-0.004	0.065	0.820	0.003	0.094	1.000	
0.050	0.058	0.063	0.011	0.079	0.781	0.022	0.106	1.000	
*0.100*	*0.089*	*0.083*	*0.036*	*0.104*	*0.729*	*0.065*	*0.133*	*0.999*	
0.150	0.114	0.097	0.063	0.133	0.694	0.102	0.155	0.999	
0.200	0.137	0.117	0.091	0.157	0.651	0.145	0.178	0.997	
m = 7, k = 4, d = 4									
0.010	0.059	0.052	0.039	0.106	0.754	0.036	0.166	1.000	
0.025	0.060	0.053	0.047	0.115	0.746	0.055	0.173	0.999	
0.050	0.064	0.064	0.065	0.133	0.744	0.079	0.181	0.999	
*0.100*	*0.085*	*0.074*	*0.098*	*0.167*	*0.707*	*0.128*	*0.195*	*0.999*	
0.150	0.095	0.089	0.129	0.196	0.687	0.173	0.210	0.998	
0.200	0.111	0.092	0.164	0.219	0.663	0.215	0.225	0.998	
m = 9, k = 3, d = 4									
0.010	0.070	0.048	0.102	0.122	0.748	0.100	0.206	1.000	
0.025	0.074	0.047	0.111	0.136	0.746	0.112	0.207	0.999	
0.050	0.073	0.056	0.128	0.157	0.735	0.137	0.213	1.000	
*0.100*	*0.092*	*0.056*	*0.160*	*0.186*	*0.710*	*0.186*	*0.222*	*0.999*	
0.150	0.094	0.067	0.192	0.213	0.695	0.229	0.232	0.999	
0.200	0.107	0.072	0.225	0.240	0.687	0.275	0.236	0.998	

Intraclass correlation, † At least one or more events in at least one cluster. Italic data corresponds to the ICC used in the design.

**Table 4:** Observed misclassification errors and estimated intraclass correlation for varying values of intraclass correlation (ICC), assuming ICC = 0.2 in the design

			**From simulations at p**_**l**_ **= 0.05**			**From simulations at p**_**u**_ **= 0.25**			
**ρ**	**α**	**β**	**ICC, mean**	**ICC, standard deviation**	**Percent of simulations with observable ICC†**	**ICC, mean**	**ICC, standard deviation**	**Percent of simulations with observable ICC**	
m = 4, k = 40, d = 20									
0.010	0.000	0.002	0.001	0.020	1.000	0.002	0.022	1.000	
0.025	0.002	0.008	0.010	0.027	0.997	0.013	0.030	1.000	
0.050	0.008	0.023	0.024	0.041	0.989	0.032	0.044	1.000	
0.100	0.029	0.056	0.047	0.064	0.960	0.070	0.070	1.000	
0.150	0.065	0.082	0.067	0.090	0.918	0.109	0.099	1.000	
*0.200*	*0.096*	*0.099*	*0.087*	*0.115*	*0.872*	*0.143*	*0.120*	*1.000*	
m = 7, k = 6, d = 6									
0.010	0.033	0.020	0.010	0.078	0.884	0.014	0.107	1.000	
0.025	0.041	0.026	0.021	0.087	0.864	0.027	0.111	1.000	
0.050	0.045	0.032	0.037	0.101	0.854	0.053	0.124	1.000	
0.100	0.066	0.046	0.071	0.132	0.825	0.098	0.141	1.000	
0.150	0.079	0.056	0.101	0.157	0.783	0.144	0.158	1.000	
*0.200*	*0.094*	*0.069*	*0.135*	*0.186*	*0.754*	*0.193*	*0.177*	*0.999*	
m = 9, k = 4, d = 5									
0.010	0.035	0.035	0.049	0.103	0.842	0.049	0.149	1.000	
0.025	0.040	0.040	0.058	0.111	0.829	0.068	0.156	1.000	
0.050	0.046	0.046	0.077	0.133	0.815	0.091	0.163	1.000	
0.100	0.058	0.052	0.113	0.163	0.800	0.139	0.177	1.000	
0.150	0.064	0.065	0.145	0.191	0.773	0.188	0.191	1.000	
*0.200*	*0.077*	*0.070*	*0.176*	*0.215*	*0.749*	*0.235*	*0.198*	*1.000*	

ICC: Intraclass correlation, † At least one or more events in at least one cluster. Italic data corresponds to the ICC used in the design.

## Discussion

Lot quality assurance sampling has already proven a valuable tool for public health program monitoring and evaluation [6]. Combining sampling in clusters and LQAS increases the versatility of the tool. However, as others have noted, failing to account for the clustering in the design of the LQAS system typically leads to inflated misclassification errors [7,8]. This paper provides the general theory for sample size calculations for C-LQAS that accounts for the clustering in the design using a beta-binomial model. We applied this theory to the CHW data quality assessments, using one set of parameters – *p*_*u*_ = 0.25, *p*_*l*_ = 0.1, α_*max*_ = 0.1 and β_*max*_ = 0.1, and ρ = 0.1. Conclusions about the design of C-LQAS parallel standard cluster sampling theory. First, for a given set of parameters, there is an interplay between the number clusters and number of individuals per cluster sampled, and sampling fewer clusters will lead to a larger total sample size. Second, if the ICC is correctly specified, or over-estimated, then the resulting C-LQAS systems will classify areas while restricting misclassification risks below the bounds set by the program manager. However, underestimating the ICC can lead to misclassification risks above the desired bounds.

### Determining the final design of the cluster-LQAS system

One challenge for C-LQAS, as with any cluster design, is determining a plausible value of ICC for the first implementation. Relevant ICC values may not be published, can change over time, and are population and design dependent. One can look to previous surveys, such as the Demographic Health Survey (DHS) for the country, to give some indication of the magnitudes of the clustering expected, especially if local information is provided [15]. In our application, the DHS does not collect data on indicators that could be directly linked to the performance of CHWs, so that option is not available. The recent CONSORT extension for cluster randomized trials requires that ICC values be reported [16]. These estimates, particularly from control groups [17], could provide insight into a range of values of ICC for the design of C-LQAS. Two studies offer insight into the ICC of CHW data quality - one study estimated the ICC of the quality of communication in nurses to be 0.28 [18] and another the median estimated ICC across a variety of quality-of-care indicators in health facilities to be 0.2 [19]. These two studies focused on quality of care, and not quality of documentation, and evaluated performance in a different group of professionals and so the relevance is limited.

As Table 1 typifies, a conservative value of the ICC is ideal to protect against high misclassification errors, although it may be a costly solution. Once the data are collected, the ICC can be estimated with confidence intervals and used for subsequent C-LQAS designs. Ridout et al. demonstrate that averaged across different settings, the ANOVA estimator has low bias and standard deviation compared to other methods to estimate ICC for binary data [14]. However, the context presented here for the assessment of CHW data quality where few clusters and few individuals per cluster are sampled, we observe large biases and standard deviations (Tables 2, 3 and 4). Most worrisome, when the fewest clusters (m = 4) are used, the ANOVA estimator is small relative to the true ρ, thus indicating a bias that would lead to underestimating the risk of misclassification. Similar results were observed by Minetti et al. for designs that had more clusters (m = 10) and few individuals per cluster [9]. Therefore, practitioners should exercise caution when choosing the best ICC value for future designs, especially if estimated from previous implementations where very few clusters were sampled.

Cost is one approach to determining the preferred design, and other factors may come into play. For example, when ρ = 0.1, the cost of the {*m = 5, k = 6, d = 4*} design is $3,000 compared to $3,500 for the {*m = 7, k = 4, d = 4*} design. For an extra $500, the latter design would still meet the misclassification constraints for larger ICCs than anticipated (up to ρ = 0.15). Further, more clusters would provide a better estimate of the ICC for future designs. Other factors such as time requirements, available human resources, geographic spread, and topography may contribute to the final decision as well.

### Limitations of the C-LQAS design

In the design of a C-LQAS system, users should be aware of several possible limitations. The sample size and decision rules, and corresponding misclassification risks, hinge on the assumed value of ICC. As discussed earlier, if the ICC is underestimated in the design, the risk of misclassification may be higher than desired. Additionally, C-LQAS is subject to the same limitations as LQAS; specifically, when many areas have true prevalences in the grey area (between *p*_*l*_ and *p*_*u*_), the survey will have very poor classification properties (commonly referred to as specificity) [9,20,21]. Prior knowledge about the underlying distribution of prevalence among the areas in the study region can be used to inform the choice of the upper and lower thresholds and the choice of design. For example, Olives and Pagano propose an alternative Bayesian approach to incorporate this prior knowledge into the design [22,23]. Additionally, the beta-binomial model assumes that there are an infinite amount of clusters in the population. When the population consists of few clusters, the classification risks presented here will overestimate the true misclassification risks. Therefore, investigators may be able to sample fewer clusters or fewer individuals per cluster than presented here while restricting misclassification risks below the specified bounds. A final limitation of the C-LQAS design presented here is the assumption that the clusters are the same size and that *m* clusters are sampled using simple random sampling without replacement. For the CHW data quality assessment, this is a reasonable assumption, as there is little variability in the number of households per village.

## Conclusion

C-LQAS has already proven useful for monitoring of neonatal tetanus eradication, vaccination coverage, and acute malnutrition [8,11,24-28]. This paper provides a probabilistic model to design C-LQAS systems in the future. We illustrate the method with the CHW data accuracy assessment in southern Kayonza, Rwanda, but the methodology presented and supplemental code are generalizable to any health program activity. Extending to other programs requires that program managers specify parameters useful for planning and evaluation – namely thresholds that meaningfully define areas that are high or low performing and allowable misclassification risks at these thresholds. Selection of these parameters is often more challenging than the determining the design once these parameters are specified as it requires consideration of program resource allocation and program objectives. Further, as highlighted in this paper, correct design of C-LQAS hinges on the assumed value of the ICC.

Along with additional evaluation of C-LQAS in the field, more methodological research is needed as well. Future work should investigate adjusting the misclassification risks to account for finite cluster sizes, accounting for varying cluster sizes, or incorporating knowledge about underlying prevalence of the trait of interest (Bayes-C-LQAS). Advances in the C-LQAS methodology will add to the set of evaluation tools that support local public health program evaluation and management, particularly in resource limited settings.

## Competing interests

The authors declare that they have no competing interests.

## Authors’ contributions

BHG led the design, analysis and interpretation of data and the drafting of the article. TM, LH, CO, and MP all made substantial contributions to the design, analysis and interpretation of data and the drafting of the article. All authors approved of the final version of the article.

## Supplementary Material

Additional file 1 Determination of number of clusters and number of individuals per cluster to sample using C-LQAS. Click here for file

Additional file 2 R code for exact sample size. Click here for file
