# Supplementary material for: The effect of clustering on lot quality assurance sampling: a probabilistic model to calculate sample sizes for quality assessments
Source: Emerg Themes Epidemiol. 2013 Oct 26;10:11. doi: 10.1186/1742-7622-10-11 (PMC3819670; doi:10.1186/1742-7622-10-11)
Supplement: Additional file 1 — Determination of number of clusters and number of individuals per cluster to sample using C-LQAS. [file 1742-7622-10-11-S1.doc]

**Appendix A: Determination of number of clusters and number of individuals per cluster to sample using C-LQAS**

After specification of the five required parameters – *pu*, *pl*,αmax, βmax, and ρ – the survey designer finds an *m*, *k*, and *d* to reduce the probability of misclassifying at the upper threshold (pu­) in the lower category below α­*max*:

$$P\left(\sum_{i=1}^{m} X_{i}<d|m,k,\rho,p_{u}\right)\leq\alpha_{\max}$$

and to reduce the probability of misclassifying at the lower threshold (pl­) in the upper category below β­*max*:

$P\left(\sum_{i=1}^{m} X_{i}\geq d|m,k,\rho,p_{l}\right)\leq\beta_{\max}$.

*Xi* represents the number of ‘defects’ observed in the random sample in cluster *i*. We directly calculate $P\left(\sum_{i=1}^{m} X_{i}|m,k,\rho,p\right)$ – the distribution of $\sum_{i=1}^{m} X_{i}$ – using

$P\left(\sum_{i=1}^{m} X_{i}|m,k,p,\rho\right)=\prod_{i=1}^{m} P\left(X_{i}|k,p,\rho\right)$.

Each Xi is an independent beta-binomial random variable, with

$P\left(X_{i}=x|k,\rho,p\right)=\left(\begin{matrix} k \\ x \end{matrix}\right)\frac{B(x+a,k-x+b)}{B(a,b)}$,

where B(.,.) is the beta function, and *a=p*(1-ρ)/ρ and *b*=(1-*p*)(1-ρ)/ρ12. Note that 0<ρ<1. Negative values for intracluster correlation are possible if individuals in the same cluster are more heterogeneous than those across different clusters, which in our application, and in most cluster sampling applications, is not plausible. Additionally, the beta-binomial model assumes that the number of clusters in the population is large relative to the number of clusters sampled; if this assumption does not hold, the sample size calculated under the beta-binomial model will be overly conservative.
