# Supplementary material for: The effect of clustering on lot quality assurance sampling: a probabilistic model to calculate sample sizes for quality assessments
Source: Emerg Themes Epidemiol. 2013 Oct 26;10:11. doi: 10.1186/1742-7622-10-11 (PMC3819670; doi:10.1186/1742-7622-10-11)
Supplement: Additional file 2 — R code for exact sample size. [file 1742-7622-10-11-S2.doc]

**Appendix B: R code for exact sample size**

The following code will create the function clqas(). To use this function, the user must type:

clqas(<value of pl­>,<value of pu>,<value of α>,<value of β>). For this command, the default maximum number of individuals per cluster is 200. To increase this amount, the user will type: clqas(<value of pl­>,<value of pu>,<value of α>,<value of β>,<value of kmax>).

The maximum number of clusters considered is equal to *nmin*. When *m=* *nmin,* only one observation per cluster is required. Any *m* larger than *nmin* will reduce the risk of errors below the desired limits. For a given *m*, if the minimum *k* has two possible decision rules that meet the necessary constraints, then the *d* that minimizes total misclassification risk is recommended.

Note that depending on the version of R and VGAM package, the code will need to change, substituting dbetabinom with dbetabin for older versions of R.

clqas<-function (pl, pu, alpha, beta, rho, k.max = 200) {

require(VGAM)

p.low <- pl

p.up <- pu

alpha.constraint <- alpha

beta.constraint <- beta

n <- c(10:1000)

results <- matrix(-5, nrow = 2, ncol = length(n))

results[1, ] <- n

for (j in 1:length(n)) {

x <- c(0:n[j])

alpha <- rep(-5, length(x))

beta <- rep(-5, length(x))

alpha.prel <- dbinom(x, n[j], p.up)

beta.prel <- dbinom(x, n[j], p.low)

alpha[1] <- 0

beta[1] <- 1

for (i in 2:length(x)) {

alpha[i] <- sum(alpha.prel[1:(i - 1)])

beta[i] <- sum(beta.prel[i:length(x)])

}

condition1 <- which(alpha < alpha.constraint)

condition2 <- which(beta < beta.constraint)

if (max(condition1) == min(condition2)) {

results[2, j] <- max(condition1) - 1

}

}

possibles <- which(results[2, ] > -1)

min.n <- results[1, min(possibles)]

cor.d <- results[2, min(possibles)]

m.max <- min.n

k.range <- c(1:k.max)

m.range <- c(2:m.max)

results <- matrix(-5, nrow <- length(m.range), ncol <- 5)

colnames(results) <- c("m", "k", "d", "alpha", "beta")

for (i in 1:length(m.range)) {

m <- m.range[i]

results[i, 1] <- m

solved <- 0

j <- 0

while (solved == 0) {

j <- j + 1

k <- k.range[j]

x <- c(0:k)

prob.x.m1.pl <- dbetabinom(x, k, p.low, rho)

prob.x.m1.pu <- dbetabinom(x, k, p.up, rho)

prob.x.all.pl <- rep(0, m * k + 1)

prob.x.all.pl[1:(k + 1)] <- prob.x.m1.pl

prob.x.all.pu <- rep(0, m * k + 1)

prob.x.all.pu[1:(k + 1)] <- prob.x.m1.pu

for (i2 in 1:(m - 1)) {

xxl <- rep(0, m * k + 1)

xxu <- rep(0, m * k + 1)

for (j2 in 0:k) {

yl <- rep(0, m * k + 1)

xstorel <- prob.x.all.pl * prob.x.m1.pl[j2 +

1]

yl[(j2 + 1):(m * k + 1)] <- xstorel[1:(m *

k + 1 - j2)]

xxl <- xxl + yl

yu <- rep(0, m * k + 1)

xstoreu <- prob.x.all.pu * prob.x.m1.pu[j2 +

1]

yu[(j2 + 1):(m * k + 1)] <- xstoreu[1:(m *

k + 1 - j2)]

xxu <- xxu + yu

}

prob.x.all.pl <- xxl

prob.x.all.pu <- xxu

}

d <- c(0:(m * k))

alpha.range <- rep(0, length(d) + 1)

alpha.range[2:length(alpha.range)] <- sapply(d, function(y) {

sum(prob.x.all.pu[1:(y + 1)])

})

beta.range <- rep(0, length(d) + 1)

beta.range[1:(length(beta.range) - 1)] <- sapply(d,

function(y) {

sum(prob.x.all.pl[(y + 1):(m * k + 1)])

})

ind <- rep(0, length(d) + 1)

ind[alpha.range <= alpha.constraint & beta.range <=

beta.constraint] <- 1

if (sum(ind) > 0)

solved <- 1

if (k == k.max)

solved <- 1

if (sum(ind) == 0) {

rule <- NA

alpha.temp <- NA

beta.temp <- NA

}

if (sum(ind) == 1) {

rule <- d[which(ind == 1)]

alpha.temp <- alpha.range[which(ind == 1)]

beta.temp <- beta.range[which(ind == 1)]

}

if (sum(ind) > 1) {

rule <- d[which((alpha.range + beta.range) ==

min(alpha.range + beta.range) & (ind == 1))]

alpha.temp <- alpha.range[which((alpha.range +

beta.range) == min(alpha.range + beta.range) &

(ind == 1))]

beta.temp <- beta.range[which((alpha.range +

beta.range) == min(alpha.range + beta.range) &

(ind == 1))]

}

}

results[i, 2] <- k

results[i, 3] <- rule

results[i, 4] <- alpha.temp

results[i, 5] <- beta.temp

}

return(results)

}
